# Diameter Dependence of Lattice Thermal Conductivity of Single-Walled Carbon Nanotubes: Study from Ab Initio

**DOI:** 10.1038/srep15440

**Published:** 2015-10-22

**Authors:** Sheng-Ying Yue, Tao Ouyang, Ming Hu

**Affiliations:** 1 Aachen Institute for Advanced Study in Computational Engineering Science (AICES), RWTH Aachen University, 52062 Aachen, Germany; 2 Institute of Mineral Engineering, Division of Materials Science and Engineering, Faculty of Georesources and Materials Engineering, RWTH Aachen University, 52064 Aachen, Germany

## Abstract

The effects of temperature, tube length, defects, and surface functionalization on the thermal conductivity (*κ*) of single-walled carbon nanotubes (SWCNTs) were well documented in literature. However, diameter dependence of thermal conductivity of SWCNTs received less attentions. So far, diverse trends of the diameter dependence have been discussed by different methods and all the previous results were based on empirical interatomic potentials. In this paper, we emphasize to clarify accurate *κ* values of SWCNTs with different diameters and in-plane *κ* of graphene. All the studies were under the framework of anharmonic lattice dynamics and Boltzmann transport equation (BTE) based on first principle calculations. We try to infer the right trend of diameter dependent thermal conductivity of SWCNTs. We infer that graphene is the limitation as SWCNT with an infinite diameter. We analyzed the thermal conductivity contributions from each phonon mode in SWCNTs to explain the trend. Meanwhile, we also identify the extremely low thermal conductivity of ultra-thin SWCNTs.

Carbon nanotubes (CNTs) are widely studied and promising for various applications due to their extraordinary electrical, thermal and mechanical properties^1^,^2^. CNTs-based material becomes one of the most attractive nanomaterials in many aspects and potential applications, e.g. reinforced composites^3^, field emission devices^4^, sensors and probes^4^, solar cells^5^, and thermal interface materials^6^,^7^,^8^. Especially CNTs have extremely high thermal conductivities (*κ*) which comes from the strong *sp*^2^ C-C bonds in perfect nanotube cylinders^9^. The value measured by experiment is 3500 W/mK for a single-walled carbon nanotube (SWCNT) with a length of 2.6 *μm* and diameter 1.7 *nm* at room temperature^10^. The molecular dynamics (MD) simulations predict the thermal conductivity as high as 6600 W/mK at room temperature^11^. Unusually high thermal conductivity makes carbon nanotube (CNT) the best promising candidate material for thermally conductive composites. Thermally conductive polymer composites offer new possibilities for replacing metal parts in several applications, including power electronics, electric motors and generators, heat exchangers, etc.^12^. It is significant and important to investigate clearly the thermal properties of CNTs from theory and experiments.

In the previous study of density functional theory (DFT), the vibrational properties of SWCNTs are studied including the radial breathing modes (RBM), tangential totally symmetric modes, zone folding approximation for phonons of nanotubes, phonon dispersion relations with the helical Brillouin zone, and the electron-phonon coupling effects^13^. Among many characteristic parameters of CNTs, the diameter and chirality directly determine their fundamental properties^14^. Therefore, it is significant to clarify the relationship between CNTs’ thermal conductivity and diameter. Classical MD simulation is an effective method to tackle such problem. However, in the MD simulations, the empirical potentials largely depend on fitting parameters from experiments and first-principle calculations. Different classical potentials in MD simulations of SWCNTs yielded diverse trends in the diameter dependence of thermal conductivity^15^,^16^. Thomas *et al*. used the second generation reactive empirical bond-order (REBO) potential to model the interactions between carbon atoms and showed that the thermal conductivity of small diameter SWCNTs is higher than that for graphene and increases with diameter descending^15^. However, using the same non-equilibrium molecular dynamics technique with different optimized Tersoff potential, Cao *et al*.^16^ demonstrated that the thermal conductivity of CNTs is generally lower than that of graphene and decreases with diameter descending. Lindsay *et al*. implemented an exact numerical solution to the Boltzmann-Peierls phonon transport equation (BTE) with phonon frequencies, eigenvectors, and third-order interatomic force constants evaluated using the optimized Tersoff potential. In their research, the *κ* of SWCNTs is smaller than graphene and exists a minimum value at a certain diameter^17^. Therefore, it is still significant to study the diameter dependance behavior of *κ* for SWCNTs and compared with graphene, which can be regarded as the SWCNT with diameter approaching infinity in structural configuration.

In this work, we applied the method which considers the anharmonic three-phonon scattering based on ab initio calculation and combines with BTE to study the *κ* of SWCNTs with different diameters. We try to investigate the high *κ* values of the SWCNTs accurately and clarify the relationship between *κ* and diameter. The calculation results agree very well with the experimental data that have been reported. From the first principle calculation results, we can infer that *κ* of SWCNTs increases with diameter decreasing, and *κ* of graphene shows to be the lowest boundary value of these SWCNTs. We also study the thermal transport in ultra-thin SWCNTs with diameters 2.07 *Å* and 2.71 *Å*. We demonstrate that the ultra-thin SWCNTs possess relatively lower thermal conductivities than graphene, which agrees with the recent MD simulations result^14^.

## Results and Discussion

**Figure 1:**
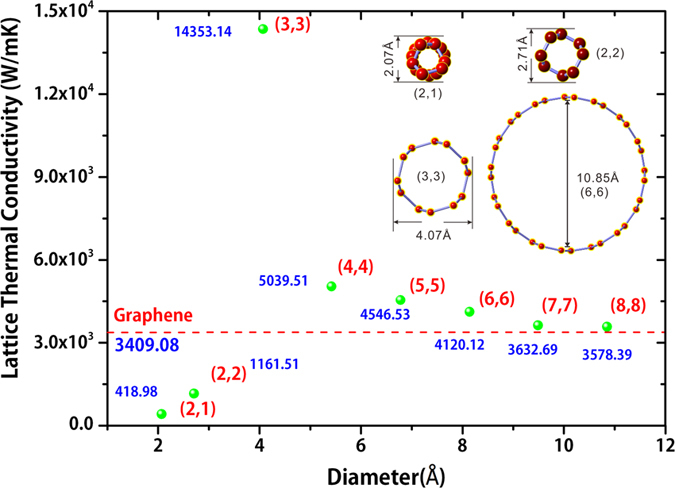
Lattice thermal conductivity of SWCNTs with different diameter. The dashed line represents the thermal conductivity of graphene. (Inset) cross-sectional view of atomic structure of typical SWCNTs.

Seven armchair SWCNTs and one chiral ultra-thin SWCNT (2,1) were calculated. In previous report, the chirality of SWCNTs does not effect *κ* obviously^17^. The absolute values of the *κ* for these SWCNTs and graphene at room temperature are displayed in Table 1. and Fig. 1. In Table 2, we displayed and compared our results of SWCNTs and graphene with results reported in literature (experimental and theoretical). Our results of SWCNTs fall in the same range of experimental measurements, i.e. from 3500 W/mK to up to 14,000 W/mK. The in-plane *κ* of graphene from our calculation is 3409.08 W/mK. This result excellently agrees with recent research by first principles^18^.

From the data in Table 1 and Fig. 1, the results indicate that the *κ* of SWCNTs increases with the diameter descending from 10.85 *Å* to 4.07 *Å*. In this diameter range, the diffusive phonon thermal conductivity *κ* of SWCNTs are larger than in-plane *κ* of graphene. From Fig. 1 we can also deduce that with the diameter increasing the *κ* along the tube direction will approach to the graphene in-plane value.

**Figure 2:**
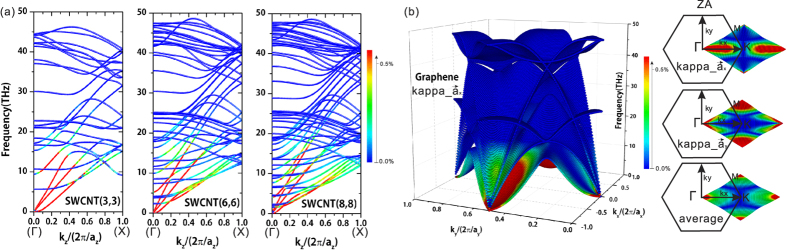
(**a**) shows the thermal conductivity contribution (in percentage) from each phonon mode of SWCNT(3,3), SWCNT(6,6), and SWCNT(8,8); (**b**) presents thermal conductivity contribution (in percentage) along lattice vector 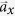 from each phonon mode of graphene in whole BZ (3D-space phonon dispersion) and phonon thermal conductivities from ZA modes (2D-projection BZ, along lattice vector 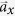, 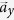, and average value of both directions).

To analyze the mechanism behind the phonon transport behavior, in Fig. 2 we plot the *κ* contribution in percentage from each phonon mode of SWCNTs and graphene. Figure 2(a) exhibits the contribution in percentage of each phonon mode to *κ* along the tube-direction for SWCNT(3,3), SWCNT(6,6) and SWCNT(8,8) respectively. Color bar indicates the contribution magnitude of each phonon mode. Red data point on phonon spectrum represents the large contribution phonon mode to *κ*. From the phonon spectrum of SWCNTs we see that there are four acoustic phonon branches and two degenerated transverse acoustic branches^19^. In phonon dispersion of SWCNT(3,3), the *κ* along tube-direction mainly originates from four acoustic phonon branches and one optical branch near the Γ point. Comparing with the phonon spectrums of SWCNT(6,6) and SWCNT(8,8), we can see that with the diameter increasing there are more optical phonon branches participating to the lattice thermal transport. These optical phonon modes concentrate on the area away from Γ point in k-space at relatively low frequency. That means there are more optical phonons participating into the optical-acoustic scattering process. This mechanism causes the total calculated *κ* in tube-direction descending with the diameter value increasing. This trend is the same with previous MD simulation study by Thomas *et al*.^15^. In Fig. 2(a) we also notice that the higher frequency optical branch which largely contributes to the tube-direction *κ* in SWCNT(3,3) becomes weaker in the SWCNT(6,6) and SWCNT(8,8). In these three phonon dispersions shown here, the phonons with frequency higher than 30 THz contribute little to the *κ*. This results is the same with the results of spectral energy density method based on MD simulation^20^.

Figure. 2(b) exhibits the each phonon mode contribution to in-plane *κ* of graphene. In the 3D phonon dispersion, we use the color to map the different contributions to the *κ* along 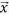 direction. And our calculation results show that graphene has a perfect isotropic property of in-plane *κ*. From the contributions in 3D phonon dispersion, we can see clearly the out-of-plane flexural (ZA) modes contribute the most to the total *κ* along 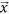. In previous research reports, ZA modes can contribute as much as 77% to of the total calculated *κ* at room temperature^21^,^22^. Simultaneously, we investigate the distributions of ZA modes *κ* contribution along 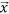, 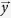 and average value of both directions in BZ. From the ZA modes contributions projecting on 2D BZ, we can observe clearly that how *κ* contributions of phonon modes distribute in two in-plane lattice directions that are equivalent in terms of geometric symmetry. And from the average *κ* contributions of ZA modes map on 2D BZ, the in-plane isotropic property is revealed clearly. In our method we calculate the in-plane phonons diffusive transport behaviors of graphene, and there do not exist the zigzag and armchair boundaries in our model. That is the origin of isotropic *κ* of graphene.

In addition, in Table 1 and Fig. 1 we find the *κ* of ultra-thin SWCNT(2,2) and SWCNT(2,1) are much lower than graphene. From cross-sectional view of their atomic structures (insert of Fig. 1), we can see that the geometric structure of C-C bands of SWCNT(2,1) and SWCNT(2,2) are quite different from other SWCNTs. The low *κ* value of the ultra-thin SWCNTs are consistent with the MD simulation results by Zhu *et al*. recently^14^.

**Figure 3:**
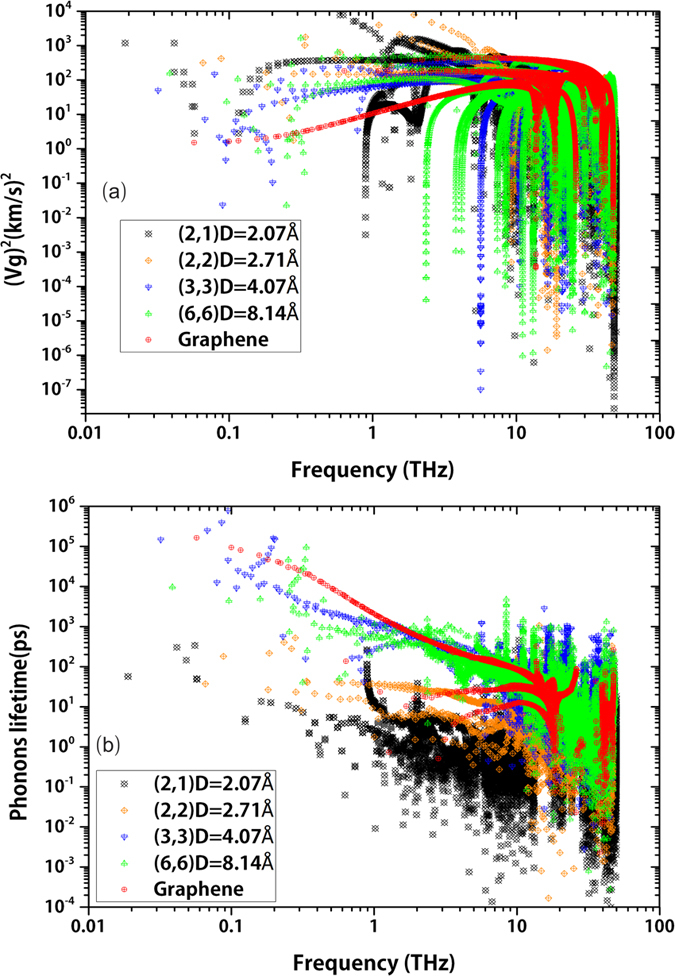
(**a**) Square of phonon group velocities with respect to frequency of SWCNTs and graphene; (**b**) Phonon lifetime with respect to frequency of SWCNTs and graphene.

In order to reveal underlying mechanism of the diameter dependence of *κ* of SWCNTs and analyze the frequency information of different SWCNTs and graphene, we calculate the factors of *κ* in Eq. (5): square of *v*_*g*_, and *τ* with respect to frequency. The results are presented in Fig. 3(a,b). In Fig. 3(a) we present the square of group velocities of each phonon with respect to frequency for SWCNTs and graphene. Combining with the phonon dispersions in Fig. 2, we can find that the four acoustic phonon branches at low frequency area of SWCNTs have the relatively higher group velocities. At the same time, these phonon modes also have the major contributions of lattice thermal conductivities along tube direction of SWCNTs. From the 3D phonon dispersion of graphene shown in Fig. 2(b), we know that the lowest one among the three low frequency group velocity lines in Fig. 3(a) belongs to the ZA modes. The result of graphene is consistent with previous study^35^. In Fig. 3(a) there exist a lot of group velocities square data points slumping at some frequencies. These small group velocities phonons come from those smooth optical branches and the smooth part of acoustic branches at high symmetry point in k-space shown in Fig. 2. The group velocity at Γ point is set to be zero.

Figure 3(b) exhibits the specific lifetime of each phonon mode from SWCNTs and graphene. The result indicates that the phonon modes in low frequency area (<10 THz) have relatively larger lifetime than high frequency area for all SWCNTs cases and graphene. And the largest lifetime of phonons (<1 THz) is about more than 10,000 times of other phonons in Fig. 3(b). Comparing with the difference of square of group velocity of phonon modes in each case, it’s easy to see the phonon lifetime is the key factor which dominates the *κ*. Figure 3(b) demonstrates the truth that long wave-length phonons have relatively small scattering in scattering processes. Figure 3(b) implies that SWCNT(3,3) has more long lifetime phonon modes than SWCNT(6,6) and graphene at the same time. This is the most distinct reason for the diameter dependence of *κ* of SWCNTs and the higher *κ* of SWCNTs than that of graphene as shown in Fig. 1.

In Fig. 3(b), we also notice that the ultra-thin SWCNT(2,1) and SWCNT(2,2) have significantly smaller phonon lifetimes than the SWCNT(3,3), SWCNT(6,6) and graphene at same frequencies. This phenomenon is reflected by the relative small *κ* as shown in Fig. 1. This result is consistent with the recent MD simulations^14^. Another information in Fig. 3(b) of the two ultra-thin SWCNTs cases is that the phonon lifetimes data points concentrate in 1 ~ 10 THz. It implies that the scattering process of optical phonons (1 ~ 10 THz) is intense in ultra-thin SWCNTs. In phonon lifetime of graphene from Fig. 3(b), we can clarify that the highest one among three low frequency (<10 THz) lines represents the ZA modes. Although the group velocities of ZA modes are smaller than LA and TA modes, ZA modes still dominate the in-plane *κ* of graphene because of the larger phonon lifetimes than other phonon modes, which have been shown in Fig. 2(b). It means that the enhancement in phonon lifetime overwhelms the reduction of phonon group velocity for graphene.

**Figure 4:**
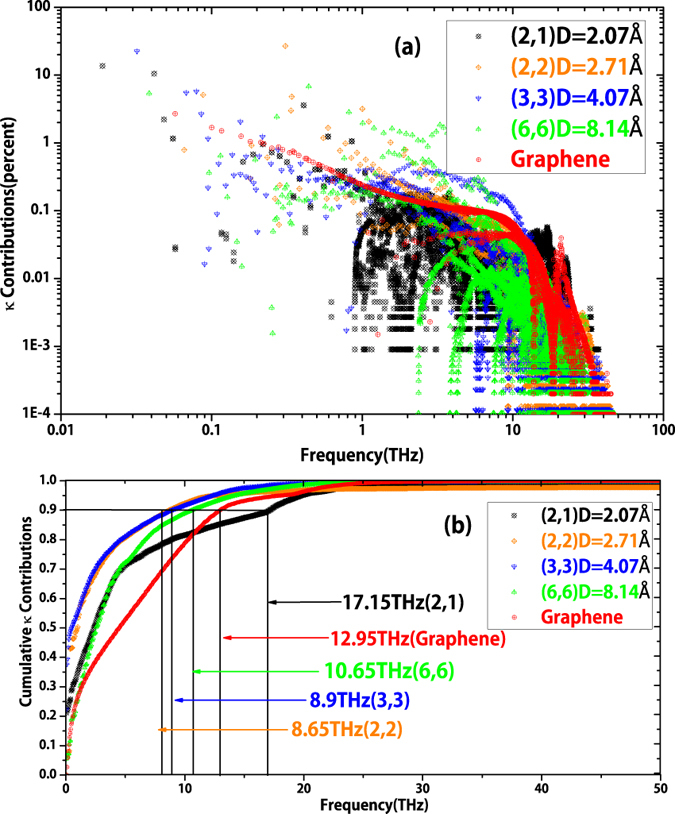
(**a**) *κ* contribution (in percentage) of each phonon mode with frequency for SWCNTs and graphene; (**b**) Accumulative *κ* contribution as functions of frequency of SWCNTs and graphene.

Summarizing the information from Fig. 3 and *C*_*ph*_, which is inversely proportional to the volume of the unit cell and descends with frequency increasing in Gaussian distribution way, we also clarify the *κ* contributions and accumulative *κ* with respect to frequency in Fig. 4. In Fig. 4(a), we can clearly identify the contributions to *κ* from specific frequencies in each case. Figure 4(b) provides again that the low frequency phonons contribute most to *κ* in SWCNTs and graphene. For the ultra-thin SWCNT(2,1) and SWCNT(2,2), the data points with very small *κ* contribution (<0.1%) concentrates in the range of 1 ~ 10 THz, due to the strong scattering among these phonons. This is consistent with our previous analysis in Fig. 3. In Fig. 4(a), we can easily distinguish the ZA modes contributions to in-plane *κ* of graphene. It indicates clearly that the contributions from ZA modes are much higher than that from other modes, and in another way reflect the same information in Fig. 2(b).

In cumulative *κ* contributions information verse frequency (Fig. 4(b)), we can learn that 90% of *κ* originates from phonons below about 10 THz for SWCNT(2,2), SWCNT(3,3) and SWCNT(6,6), while for graphene, 90% of in-plane *κ* originates from phonons below 12.95 THz. However, for SWCNT(2,1) because the much more optical phonon modes each one carries a little contribution to *κ*, the 90% contribution threshold is increased to 17.15 THz.

## Conclusion

In conclusion, we studied the lattice thermal conductivities of seven armchair SWCNTs from (2,2) to (8,8) and one chiral ultra-thin SWCNT(2,1) through the lattice anharmonic dynamic coupled with BTE method based on the first principle calculations. We also provide the in-plane *κ* of graphene for comparison with the same method. Our results suggest that lattice thermal conductivity of SWCNTs increases with diameter descending and graphene behaves as lower limit of SWCNTs with infinite diameter. We also indicate that the ultra thin SWCNTs have relatively small *κ*. From the analysis of each phonon mode contribution to overall *κ* (phonon polarization), we clarify that the SWCNT with larger diameter has more optical phonons participating in the scattering process, leading to the reduced *κ* with diameter increasing, and finally approaching the graphene limit. For graphene, by analyzing phonon mode specific group velocity and phonon lifetime, we observe the dominant role of ZA modes, originating from the overwhelming enhancement in their phonon lifetime compensating the low phonon group velocity. However, due to the limitation of the computing resource and the huge computational demand, the *κ* of SWCNTs with larger diameters do not exhibit here. More calculations are still needed to reveal the real trend about the *κ* behavior of SWCNTs with much larger diameters. Nevertheless, we expect that our work provides deeper insight into the phonon transport mechanism in small diameter carbon nanotubes and offers a clear and reliable trend for device applications involving thin carbon nanotubes in the future.

## Theory and Method

Thermal conductivity is introduced through Fourier′s law^36^:


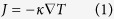


where *J* is the net heat flux, *κ* is the thermal conductivity tensor^37^. *J* can also be expressed as:


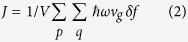


where *V* is the system volume, *p* and *q* denote the phonon branch and wave vector respectively, *v*_*g*_ is the phonon group velocity and *δf* is the net diffusive change of phonon distribution. In steady state, the rate of phonon distribution total change is zero. This condition renders the BTE form:


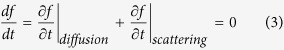


where the diffusive term can be treated as linear with the gradient of temperature:


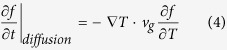


Combining Eqs (1), (2) and (4) under the relaxation time approximation it yields an expression for the thermal-conductivity tensor^38^,


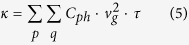


where *τ* is the phonon lifetime, 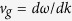, and *C*_*ph*_ is the specific heat capacity of system:


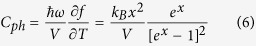


where 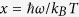. For the thermal conductivity tensor of SWCNTs, we only consider the element 
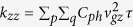
, and for graphene we calculate the diagonal elements 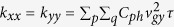
39. Phonon properties including frequencies, velocities and scattering rates are largely determined by interatomic force constants (IFCs)^40^. Unlike nanowires, SWNTs do not have an outer surface to provide boundary scattering. Therefore, for phonons traveling through a perfect nanotube, the only scattering mechanisms are due to lattice anharmonicity, and 3-phonon scattering processes taking the dominant part^41^. We can obtain *C*_*ph*_ and *v*_*g*_ from the harmonic phonon dispersion calculated by the second order IFCs, and get the scattering rate 1/*τ* from the third order IFCs^40^.

Both second and third IFCs were obtained from the first-principle calculations. Before the IFCs calculations we optimized the structures of each case in unit cell. All first-principles calculations were performed based on the density functional theory (DFT) as implemented in the Vienna Ab-initio Simulation Package (VASP)^42^,^43^. The Perdew-Burke-Ernzerhof (PBE) of generalized gradient approximation (GGA) was chosen as the exchange correlation functional^44^. We used projector augmented wave potentials^45^,^46^ to describe the core (1*s*^2^) electrons, with the 2*s*^2^ and 2*p*^2^ electrons of carbon considered as valence electrons. The kinetic energy cutoff of wave functions was set as 500 eV, and Monkhorst-pack k-mesh 4*4*24 including Γ point was used to sample the Brillouin Zone (BZ). A vacuum layer 10 *Å* was used to hinder the self-interactions between cylinders arising from the employed periodic boundary condition. All geometries were fully optimized with the Hellmann—Feynman force tolerance is 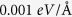. In the calculation of phonon dispersion, the supercell (1*1*6) was constructed. And the convergence of length was examined. Because of we only considering the phonons diffusive transport behaviors here, the length of SWCNTs only needs to be longer than the interactive cutoff distance between atoms theoretically. The harmonic second order IFCs were obtained within the linear response framework by employing the density functional perturbation theory (DFPT) as implemented in the VASP code^42^. Then we obtained the phonon dispersion using the PHONOPY package^47^ based on the harmonic second order IFCs.

For the calculation of *κ*, anharmonic third order IFCs are also necessary. The same supercell and k-mesh were used to get the anharmonic third order IFCs, and interactions between atoms were taken into account up to forth nearest neighbors. The total number of third order IFC evaluations depends on the number of carbon atoms in the supercell. With the third order IFCs, we solved the phonon BTE with iterative method with ShengBTE code developed by Li *et al*^48^. We calculated the final scattering rate of each phonon mode 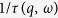 through this program. Finally using Eq. (5), we obtained the final *κ*. The grid convergence in k-space of all cases were examined^48^ and all the *κ* reported here was the converged value (for example, the k-space sampling grid of graphene was 100*100*1 as input parameter for ShengBTE). In details for ShengBTE calculations of SWCNTs and graphene, the interactive length we took is 3.4 *Å* which is the van der Waals force radius of carbon atoms. For the SWCNTs with diameter longer than 3.4 *Å*, we used the cross-sectional area^1^ *S* = *πdh*, where *d* is the diameter of SWCNT and *h* = 3.4 *Å*. In ultra-thin SWCNTs which their diameters shorter than 3.4 *Å*, the cross-sectional area we used was 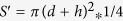 (van der Waals interaction was not in consider here).

## Additional Information

**How to cite this article**: Yue, S.-Y. *et al*. Diameter Dependence of Lattice Thermal Conductivity of Single-Walled Carbon Nanotubes: Study from Ab Initio. *Sci. Rep*. **5**, 15440; doi: 10.1038/srep15440 (2015).

## Figures and Tables

**Table 1:** *κ* of SWCNTs with different diameter and graphene at 300 K.

Sample	*κ*(*Wm*^−1^*K*^−1^)	Diameter	Chirality
*SWCNT*	418.98	2.07 *Å*	(2,1)
*SWCNT*	1161.51	2.71 *Å*	(2,2)
*SWCNT*	14353.14	4.07 *Å*	(3,3)
*SWCNT*	5039.51	5.42 *Å*	(4,4)
*SWCNT*	4546.53	6.78 *Å*	(5,5)
*SWCNT*	4120.12	8.14 *Å*	(6,6)
*SWCNT*	3632.69	9.49 *Å*	(7,7)
*SWCNT*	3578.39	10.85 *Å*	(8,8)
*Graphene*	3409.08	∞	—

**Table 2:** *κ* of SWCNTs and graphene in this work and previous experimental and theoretical research at room temperature.

Sample	*κ*(*Wm*^−1^*K*^−1^)	Method	Comments	Refs
*SWCNT*	3580–14350	BTE and ab-inito	Diffusive	This work
*SWCNT*	3270–9800	Measurement:Heater sensor	Neglected boundary resistance	Yu *et al*.^23^
*SWCNT*	~3,500	Electrical self-heating	Individual; boundary	Dai *et al*.^10^
*SWCNT*	1,750–5,800	Thermocouples	Bundles; diffusive	Hone *et al*.^39^
*SWCNT*	3,000–7,000	Electrical; micro-heater	Individual; ballistic; suspended	Yu *et al*.^23^
*SWCNT*	3160	4-pad 3*ω*	Neglected boundary resistance	Wang *et al*.^24^
*SWCNT*	3210	4-pad 3*ω*	Neglected boundary resistance	Wang *et al*.^25^
*SWCNT*	~2,500	BTE	*K*_*CNT*_ < *K*_*G*_	Mingo *et al*.^17^
*SWCNT*	~7,000	Molecular dynamics and BTE	*L* > 20 *nm*	Donadio *et al*.^26^
*Graphene*	~2,000–5,000	Raman optothermal	Suspended; exfoliated	Balandin^27^ & Ghosh^28^
*Graphene*	~2,500	Raman optothermal	Suspended; CVD	Cai *et al*.^29^
*Graphene*	1,500–5,000	Raman optothermal	Suspended; CVD	Jauregui *et al*.^30^
*Graphene*	2,000–5,000	Valence force field, BTE, *γ*(q)	Strong width dependence	Nika *et al*.^31^
*Graphene*	1,000–5,000	Relaxation-time approximation, *γ*_*TA*_,*γ*_*LA*_	Strong size dependence	Nika *et al*.^32^
*Graphene*	8,000–10,000	Molecular dynamics, Tersoff	Square graphene sheet	Evans *et al*.^33^
*Graphene*	1,400–2,400	Boltzmann transport equation	Length dependence	Lindsay *et al*.^17^
*Graphene*	~4,000	Ballistic	Strong width dependence	Muñoz *et al*.^34^
*Graphene*	~3400	BTE and ab-inito	Diffusive in-plane	This work
